# Contribution of Polyphenol Oxidation, Chlorophyll and Vitamin C Degradation to the Blackening of *Piper nigrum* L.

**DOI:** 10.3390/molecules23020370

**Published:** 2018-02-09

**Authors:** Fenglin Gu, Feifei Huang, Guiping Wu, Hongying Zhu

**Affiliations:** 1 Spice and Beverage Research Institute, Chinese Academy of Tropical Agricultural Sciences (CATAS), Wanning 571533, China; xiaogu4117@163.com (F.G.); gd_xiaogu@163.com (F.H.); guiping81@163.com (G.W.); 2 National Center of Important Tropical Crops Engineering and Technology Research, Wanning 571533, China; 3 Key Laboratory of Genetic Resources Utilization of Spice and Beverage Crops, Ministry of Agriculture, Wanning 571533, China

**Keywords:** phenol oxidation, chlorophyll, vitamin C, blackening, *Piper nigrum* L.

## Abstract

Black pepper (*Piper nigrum* L.) is the most widely used spice in the world. Blackening is considered to be beneficial and important in the processing of black pepper because it contributes to its color and flavor. The purpose of this paper is to investigate polyphenol oxidation as well as the chlorophyll and vitamin C (VC) degradation in the blackening of *Piper nigrum* L. Black pepper was produced by four methods, and changes in polyphenols, chlorophyll and VC were studied by high performance liquid chromatography (HPLC) and ultraviolet-visible and visible (UV-Vis) spectrophotometry. The results show that polyphenol oxidase activity significantly decreased during the preparation of black pepper, and the concentrations of phenolic compounds, VC, and chlorophyll a and b also significantly decreased. Polyphenol oxidation and chlorophyll and VC degradation contribute to the blackening. A crude extract of phenolic compounds from black pepper was prepared by the system solvent method. The greater the polarity of the extraction solvent, the higher the extraction rates of the phenolic compounds and the total phenol content. Pepper phenolic compounds were analyzed by HPLC analysis.

## 1. Introduction

Pepper (*Piper nigrum* Linnaeus) is an important commercial spice that is valued for its pungency and flavor. Black, white, and green pepper are the three main types of pepper products on the market. Black pepper (*Piper nigrum* L.) is an important health food and the most widely used spice in the world. Blackening is considered to be beneficial and important in the processing of black pepper, contributing to the color and flavor of black pepper.

The blackening of pepper involves enzymatic and non-enzymatic reactions. Polyphenol oxidase (PPO) and peroxidase (POD) are the primary enzymes that cause browning, and the most important is PPO. PPO can catalyze two different reactions: hydroxylation of monohydric phenols to produce the corresponding *o*-dihydroxy compounds and *o*-diphenol oxidation, which leads to the formation of *o*-benzoquinone. Both reactions are aerobic. Variyar et al. reported that the blackening of fresh green pepper berries was due to the enzyme-catalyzed oxidation of 3,4-dihydroxy phenylethanol glycoside by *o*-diphenol oxidase present in the fruit, and the enzyme was also found to be active towards the aglycone of 3,4-dihydroxy phenylethanol glycoside [1]. However, Gu et al. [2] found heat treatment deepened the color of the pepper berry, but the activity of the PPO was very low. Therefore, there could be other blackening pathways besides enzymatic reactions.

The major chlorophylls in foods include chlorophyll a and b. Chlorophyll a degrades into chlorophyll derivatives (pheophytin a, pyrophyllium chlorophyll a and pheophorbide a) [3,4], which is one of the important ways to participate in browning because chlorophyll a is sensitive to light, heat and pH. The degradation of chlorophyll a may fit a first-order model under either heating or illumination treatment [5]. Non-enzymatic browning reactions are often associated with vitamin C (VC) during the processing and storage of fruit and vegetable products. VC is oxidized into carbonyl-containing compounds, followed by reactions with other substances and/or polymerization [6].

In the current study, we analyzed the phenolic compounds as well as chlorophyll and VC degradation during the preparation of four black pepper samples. The black pepper samples preparation were grouped into four methods according to specific treatments: direct sun drying, blanching before sun drying, one-day sun drying before mild fermentation, and two-day sun drying before mild fermentation. We explored the blackening mechanisms in these four sample groups and speculated about the possible contribution of the phenolic substances to the blackening. 

## 2. Results and Discussion

### 2.1. Changes in PPO Activity

**Figure 1:**
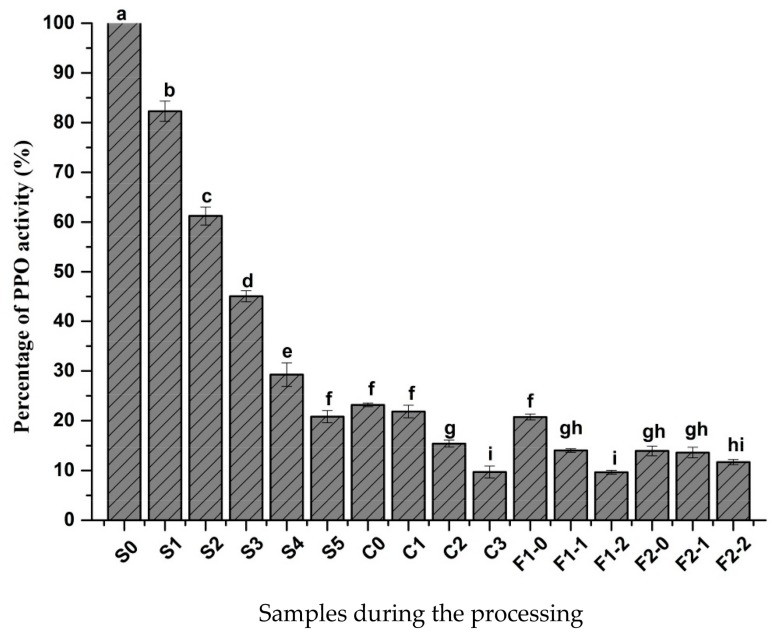
Changes in the relative polyphenol oxidase activity during the preparation of black pepper. Different letters (a, b, c, and so on) on the columns indicate significant differences between the samples (*p* < 0.05). Data are presented as means ± standard error of mean. The meaning of S0, S1, and other samples is provided in Table 1.

Black pepper was processed by four methods according to the specific treatments, and three of the methods incorporated a pretreatment of blanching or fermentation. As shown in Figure 1, compared with fresh fruit, PPO activity reduced to 23.17% after blanching. This result was lower than that reported in a previous study [2]. PPO in pepper has poor thermostability, which is similar to the characteristic of PPO in various crops. Therefore, heat treatment is commonly carried out in food processing for green color protection. The relative activity of PPO decreased significantly (*p* < 0.05) after the direct sun drying process, and this was in response to a loss of water and high temperature. The mild fermentation treatment slightly influenced the activity of PPO. The relative activities of PPO in the four final products of S5, C3, F1-2 and F2-2 were 20.83%, 9.70%, 9.65%, 11.71%, respectively. PPO had certain activity in the whole process until the final product, and therefore the PPO of samples with blanching also participated in the browning during processing. Krissana et al. analyzed the seed browning of hot peppers and found that browning was positively correlated with higher initial activities of PPO [7]. Variyar et al. found that the blackening of fresh green pepper berries was due to the enzyme-catalyzed oxidation of 3,4-dihydroxy phenylethanol glycoside by an *o*-diphenol oxidase which was also found to be active toward the aglycone of 3,4-dihydroxy phenylethanol glycoside [1]. Pruthi et al. reported that the green pepper samples were blanched in order to hinder this enzyme action and then dried in a cross-flow drier [8]. Therefore, the contribution of PPO to the blackening of pepper berries after blanching needs further examination.

### 2.2. Changes in Total Phenolic Content

**Figure 2:**
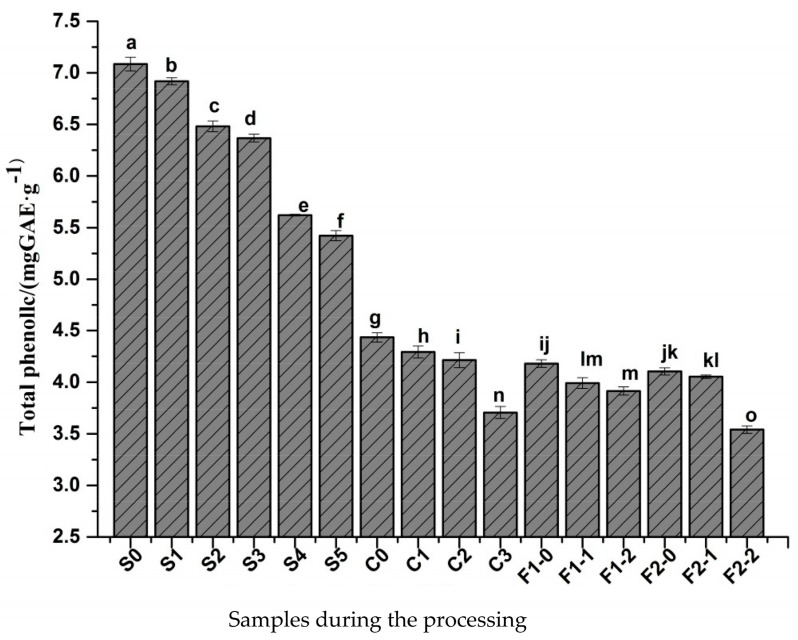
Changes in the total phenolic content during the preparation of black pepper. Different letters (a, b, c, and so on) on the columns indicate significant differences between the samples (*p* < 0.05). Data are means ± standard error of mean. The meaning of S0, S1, and other samples is provided in Table 1.

The changes in total phenolic content in pepper samples that underwent four different treatment methods were studied (Figure 2). The total phenolic content decreased significantly from 7.08 mg/g to 5.42 mg/g after direct sun drying. High temperature sun and low water content lead to polyphenol loss, which has been reported in other crops [9]. After the blanching treatment, the total phenolic content decreased by 37.43%, showing that heat treatment of 80 °C for 120 s caused significant loss of phenolic compounds. The heat treatment damaged the cell structure, altering the interaction between PPO and phenolic compounds. As a result, enzymatic browning occurred [1]. 

It is also possible that some polyphenols were lost when the berries were blanched. In this study, we observed that the black pepper was blacker in color and more uniform after the heat treatment than the direct sun drying. As to the samples of the after one-day sun drying before mild fermentation and two-day sun drying before mild fermentation, the total phenolic content decreased by 2.56% and 15.9%, respectively. The difference between them was that the water content after two days of mild fermentation was lower. Thus, the water content affected the total phenolic content, and the mild fermentation treatment promoted the release of phenols from the cell wall [10]. Bandyopadhyay et al. found that phenolic compounds decrease by 75% and phenolic compounds oxidized by *o*-diphenol oxidase completely disappeared in naturally dried pepper berries. These findings indicate that the phenolic compounds are a major contributing factor in the production of black pepper. The researchers also obtained 3,4-dihydroxy-6-(*N*-ethylamino) benzamide, the major substrate of *o*-diphenol oxidase, via separation but failed to determine the relationship between the amount of phenolics oxidized by *o*-diphenol oxidase and the degree of blackening. Therefore, the blackening of the pepper berries may involve other mechanisms [11].

### 2.3. Separation of Pepper Phenolic Compouds

#### 2.3.1. Phenolic Compounds in Black Pepper Extracts

**Figure 3:**
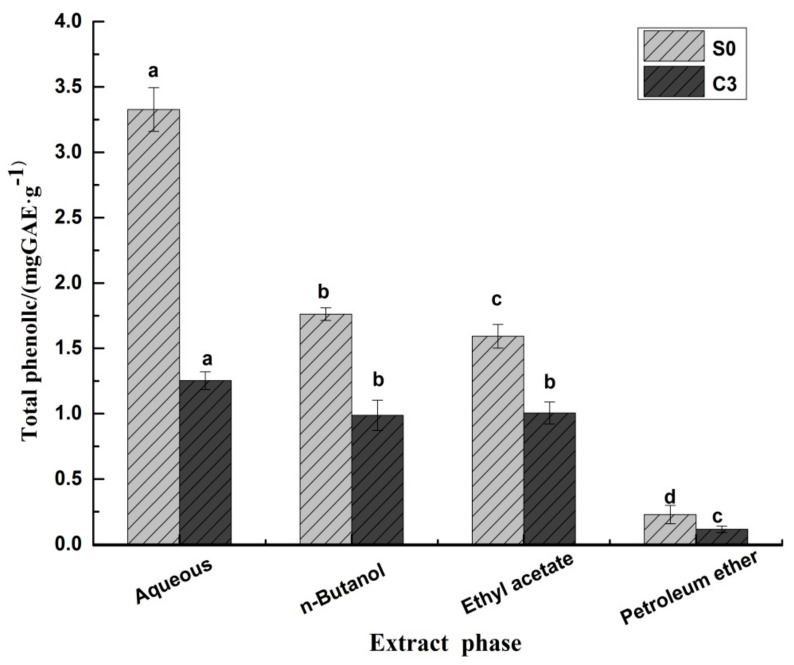
Total phenolic content after fractional extraction of pepper of S0 and C3. Different letters (a, b, c, and so on) on the columns indicate significant differences between the four extracts (*p* < 0.05). Data are means ± standard error of mean.

A crude extract of pepper phenols was extracted by system solvent method (Figure 3). Phenols were extracted by water, *n*-butanol, ethyl acetate and petroleum ether. The total phenolic content of fresh pepper (S0) in the aqueous phase was 3.33 mg GAE/g (gallic acid equivalents per g). The total phenolic content in pepper samples that underwent sun drying after blanching (C3) was 1.25 mg GAE/g. The total phenolic content of S0 in the *n*-butanol phase was 1.76 mg GAE/g, and the total phenolic content of C3 was 0.987 mg GAE/g, the phenol content of S0 in ethyl acetate phase was 1.59 mg GAE/g, the total phenolic content of C3 was 1.00 mg GAE/g, and the total phenolic content of S0 in petroleum ether was 0.23 mg GAE/g, the total phenolic content of C3 was 0.16 mg GAE/g. Compared with the total phenolic content in the four solvents of fresh fruit (S0), the content of phenolic compounds of C3 in the aqueous phase decreased to 62.35%, followed by the petroleum ether phase (49.23%), *n*-butanol phase (43.98%), and ethyl acetate (36.89%). Chatterjee et al. found that total phenolic content of green pepper, as estimated by the Prussian blue method, was found to be 850 mg catechin equivalent per 100 g of green pepper; the quantity is the same as the total phenolic content of the four extracts combined [12]. The crude extract of pepper phenolic compounds of C3 may be the “enzyme inactive” group of pepper phenolic acids as referred by Variyar and Bandyopadhyay, who reported that the “enzyme inactive” group of pepper comprised nine phenolic acids, namely, protocatechuic, gentisic, *p*-hydroxy benzoic, vanillic, caffeic, syringic, ferulic, synapic and salicylic acids. All these phenolic compounds are easily extracted by high polarity solvents [13].

#### 2.3.2. HPLC Analysis

**Figure 4:**
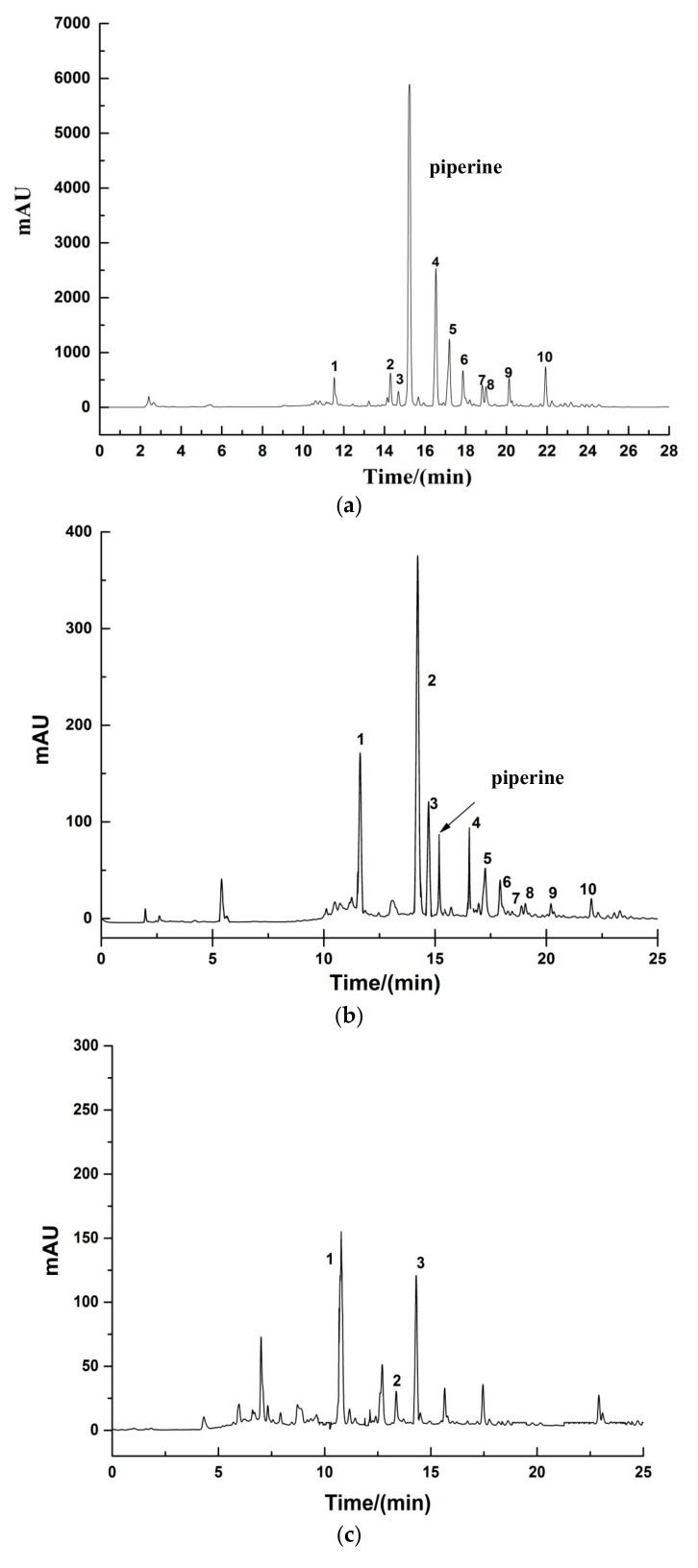

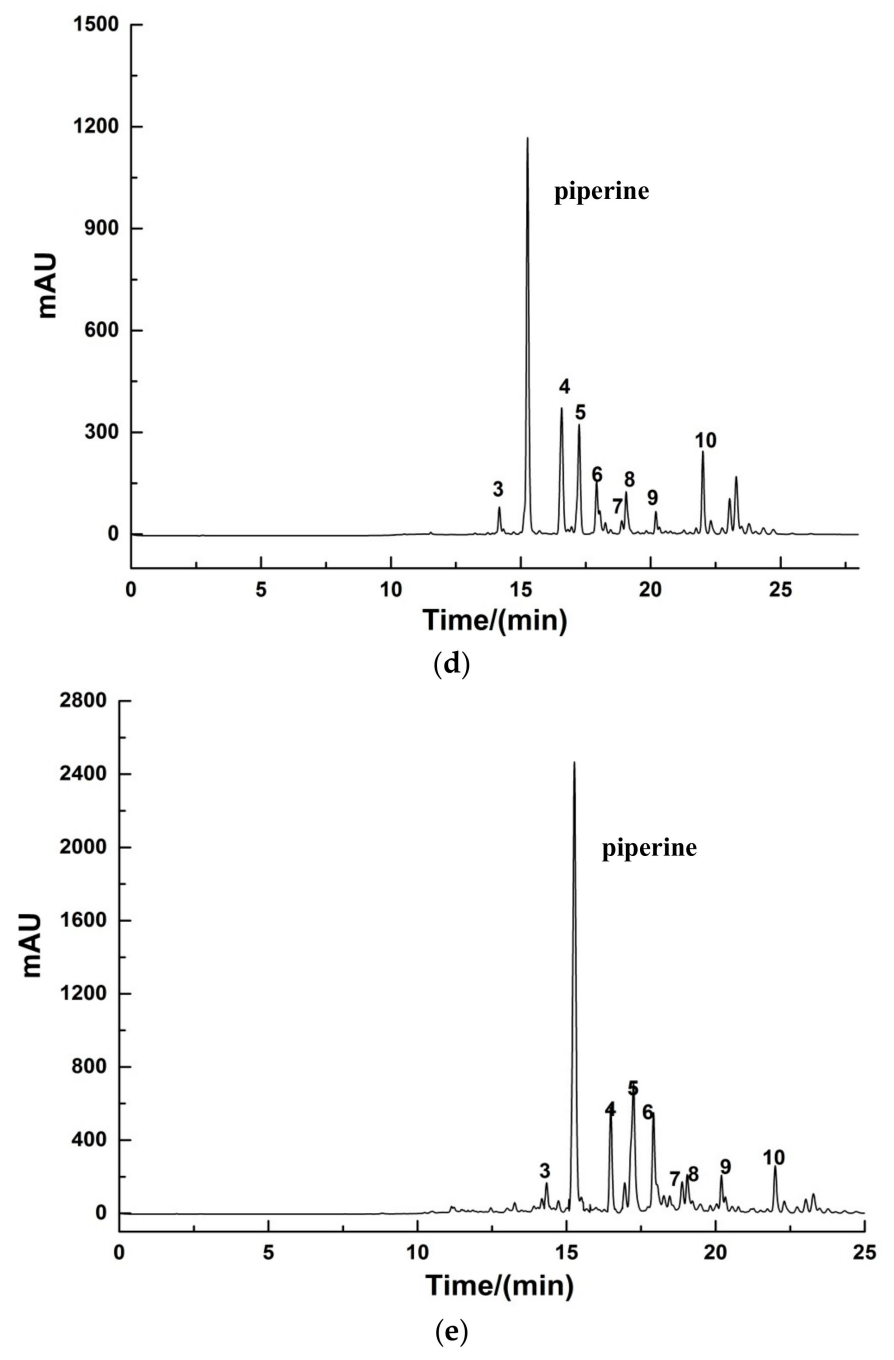
HPLC profiles of pepper phenolic crude extracts of S0 (**a**) and C3 pepper phenolic fractional extracts: (**b**) aqueous extracts; (**c**) *n*-butanol; (**d**) ethyl acetate; (**e**) petroleum ether.

A gradient HPLC method was developed for the determination of phenolic compounds in pepper samples. As shown in Figure 4, 10 peaks were baseline resolved, with excellent peak shapes. Figure 4 compared the peak area of 10 peaks in black pepper samples that underwent four different treatments with that of fresh pepper, and the peak areas were significantly different (*p* < 0.05), with the exception of peak six.

Polar compounds eluted first from the reversed-phase column. From Figure 4, the phenolic compounds in the aqueous phase were mainly concentrated in the first 15 min. Piperine and other compounds eluted after 15 min, with low response values. The phenolic compounds in the *n*-butanol phase were concentrated between 12 and 22 min. Phenolic compounds in ethyl acetate phase eluted mainly after 15 min. The phenolic compounds in the petroleum ether phase were similar to those of ethyl acetate, but the peak areas of compounds in the petroleum ether phase was greater than that of the ethyl acetate phase. In this experiment, the phenolic compounds extracted by four kinds of solvents in pepper samples have been separated by HPLC, but the specific phenolic compounds were not confirmed, and even so, the HPLC analysis methods and results could be used for qualitatively and quantitatively determining the phenolic compounds in further research. 

### 2.4. Changes in Chlorophyll a and Chlorophyll b Content 

**Figure 5:**
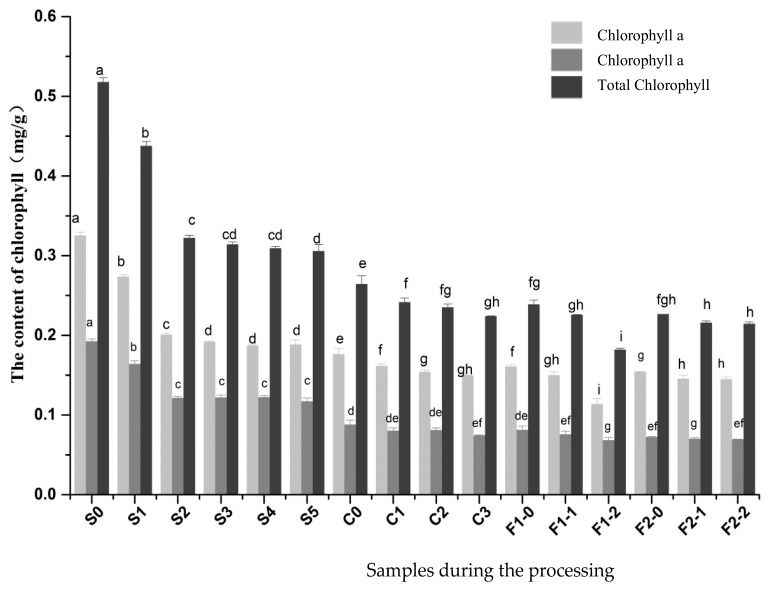
Chlorophyll content of pepper samples prepared by four different methods. Different letters (a, b, c, and so on) on the columns indicate significant differences between the samples (*p* < 0.05). Data are expressed as means ± standard error of mean. The meaning of S0, S1, and other samples are provided in Table 1.

As shown in Figure 5, the content of chlorophyll a and chlorophyll b in fresh pepper berries were 0.33 mg/g and 0.19 mg/g, respectively. The content of chlorophyll a was 2–3 times more than chlorophyll b in many green plants [10]. After the blanching treatment, the content of chlorophyll a in the pepper berries was reduced to 0.18 mg/g. The content of chlorophyll a and chlorophyll b decreased significantly as a result of blanching, in which chlorophyll was dissolved in the plant and transformed to pheophytin after heat treatment, leading to a color change from green to brown [3]. The degradation rate of chlorophyll b was 12.44% higher than chlorophyll a for the blanching treatment group, which may be related to the different degradation pathway. Chlorophyll b transformed into chlorophyll a during chlorophyll degradation, and chlorophyll a produced various derivatives [4].

The results also showed that chlorophylls degraded on the first day and the second day (*p* < 0.05), and then the degradation was not obvious. Chlorophyll a degraded into chlorophyll derivatives (pheophytin a, pyrophyllium chlorophyll a and pheophorbide a), which are involved in pepper browning. This important aspect mainly occurred in the early stage during the processing. The chlorophyll a and chlorophyll b in black pepper degraded during the mild fermentation, which was related to the change of pH during the fermentation process. It has been reported that weak acids promote chlorophyll degradation [4].

### 2.5. Changes in VC Content 

**Figure 6:**
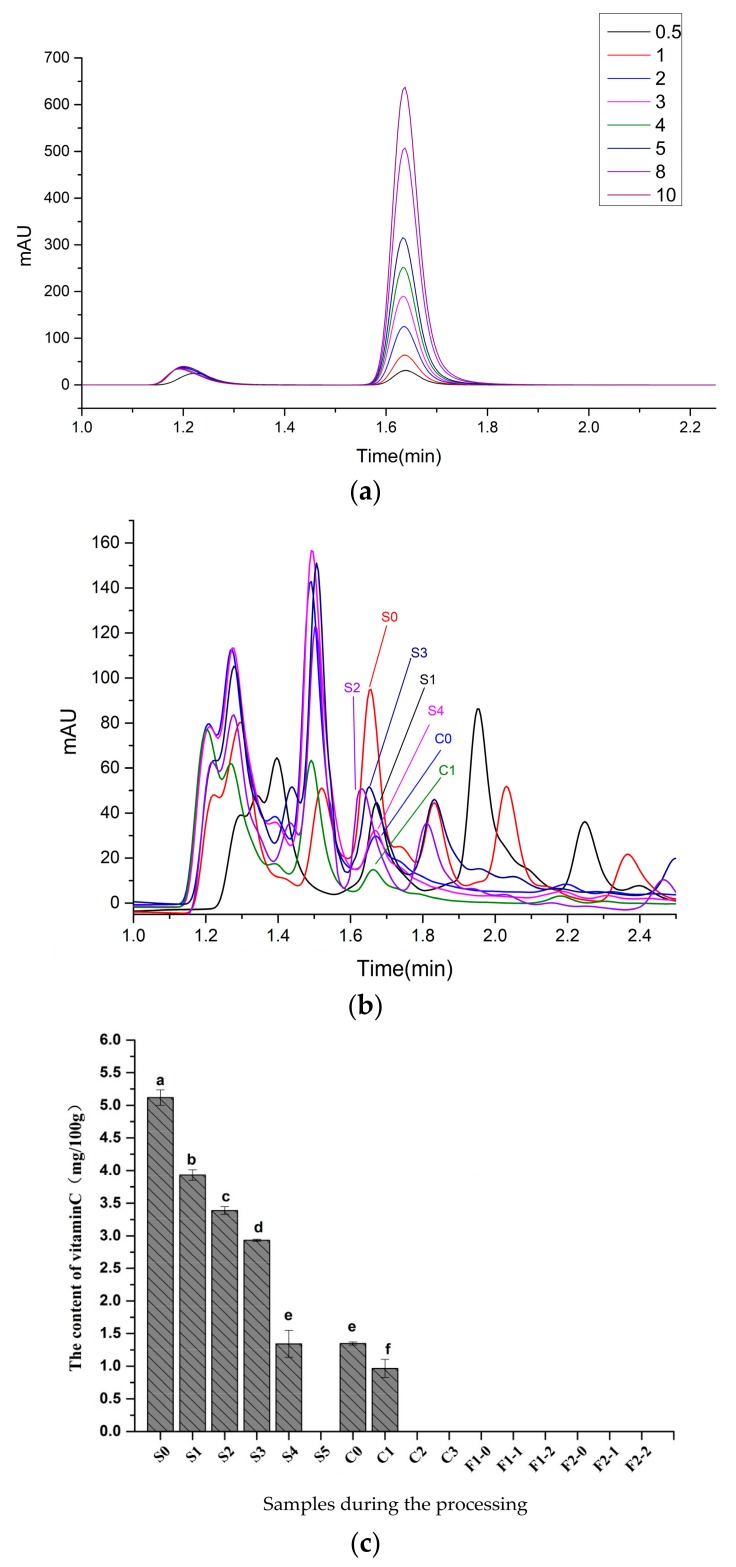
The HPLC chromatograms of a VC standard (**a**) and pepper samples (**b**). Bar graph of the VC content of pepper samples prepared by four different methods (**c**). Different letters (a, b, c, and so on) on the columns indicate significant differences between the samples (*p* < 0.05). Data are means ± standard error of mean. The meaning of S0, S1, and other samples are provided in Table 1.

The HPLC chromatograms of a VC standard and samples are shown in Figure 6a,b. The VC content of black pepper was determined by HPLC, and it was 5.12 mg/100 g in the fresh fruit (Figure 6c). In the process of direct sun drying, VC content decreased by 1.18 mg/100 g on the first day and then reduced by 0.54 mg/100 g and 0.46 mg/100 g, respectively. VC content reduced by 1.59 mg/100 g and 1.34 mg/100 g on the fourth and fifth days, respectively. Natural light can promote VC degradation, resulting in oxidation reactions. The degradation of VC in the late stage during the processing was also related to low water content, as reported by Herbig and Renard [14]. The higher the water content, the slower the degradation of VC will be.

After the blanching treatment, the VC content decreased to 1.35 mg/100 g, with a loss of 73.67%, and the reason was that high temperature damaged VC [15]. The loss of VC in the four treatment groups was substantial, and VC could not be detected by the experimental method. In the blanching of hot peppers (*Capsicum annuum* L.), VC content decreased progressively as blanching conditions were more severe (higher temperature and longer treatment duration). The VC content decreased to about 45% and 30% of the initial value, for green and red peppers, respectively. Differences in VC retention may be attributed to differences in genetics, maturity stage, brine composition, blanching method, as well as treatment time and temperature. These factors can cause different degrees of inactivation of ascorbic acid oxidase and removal of residual oxygen from vegetable tissue [14].

## 3. Experimental Section

### 3.1. Materials

Pepper berries were harvested from the plant garden of the Spice and Beverage Research Institute at the Chinese Academy of Tropical Agricultural Sciences in China. All chemical reagents were of analytical grade and chromatographic grade. HPLC grade methanol was purchased from Fisher Scientific (Fair Lawn, NJ, USA), and HPLC-grade acetonitrile and reagent grade formic acid were obtained from Merck KGaA (Darmstadt, Germany). Distilled water was purified with Milli-Q Integral System Millipore (Bedford, MA, USA). Gallic acid and other chemicals were purchased from Sinopharm Chemical Reagent Co., Ltd. (Shanghai, China).

### 3.2. Preparation of Black Pepper

Black pepper was treated by the four methods presented in Table 1. Treatment was carried out until the moisture content was 8–12%. The fermentation process was carried out by bagging with sealed plastic bag and stayed at room temperature for natural fermentation. The samples were analyzed every 24 h during the process. The dried samples were lyophilized using a vacuum freeze dryer. Samples were crushed and passed through a 60 mesh sieve. Three replicates were analyzed per treatment.

### 3.3. Preparation of Phenolic Extracts

Phenolic compounds were extracted according to a previously described method with slight modifications [12]. Each of these samples (5 g dry weight) was individually extracted with 80% aqueous methanol (50 mL) under reflux for 45 min. Reflux was continued for 1 h and the contents were then extracted with 80% aqueous methanol (20 mL × 2) in 100 mL volumetric flasks in an Omnimixer until the extracts were pale yellow. These samples were used for the determination of total phenolic content. The rest of S0 was concentrated in vacuo for HPLC analysis. S0 and C3 were extracted according to the above method.

### 3.4. Determination of Total Phenolic Content

The total phenolic phytochemical concentration was measured using the Folin-Ciocalteu method with a slight modification [16]. Briefly, 0.5 mL of appropriately diluted samples and a standard solution of gallic acid were added to a 25 mL volumetric flask. A reagent blank using double distilled H_2_O (ddH_2_O) was prepared. To the mixture, 2 mL Folin-Ciocalteu phenol reagent (10%) were added, and the mixture was shaken. After 1 min of shaking, 3 mL of a 10% Na_2_CO_3_ solution were added with mixing. The solution was then immediately diluted to a volume of 25 mL with ddH_2_O and mixed thoroughly. After incubation for 40 min at 28 °C, the absorbance of the solution, relative to that of a prepared blank, was measured at 760 nm using a spectrophotometer. The total phenolic contents of the samples are expressed in mg per serving of gallic acid equivalents (GAE). All samples were prepared in triplicate.

### 3.5. Solvent Extraction

The phenolic extracts of S0 and C3 (100 mL) were successively extracted with ultrapure water (5 × 20 mL), diethyl ether (5 × 20 mL), ethyl acetate (5 × 20 mL), and *n*-butanol (10 × 20 mL), and the solvent was removed by rotary evaporation between the two steps. Each extract was dried by rotary evaporation and reconstituted in 80% methanol to 100 mL volumetric flasks, before the determination of total phenolic content and HPLC analysis.

### 3.6. High Performance Liquid Chromatography (HPLC)

After filtration with No.4 Whatman filter paper, the phenolic extracts of C3 were given a final filtration (0.45 μm) before HPLC analysis. An HPLC system (Agilent 1260, Palo Alto, CA, USA) equipped with a quaternary pump, an inline degasser, a thermostatic autosampler, and a diode array detector (DAD) was used for the identification and quantification of various phenolic compounds in the samples. An Eclipse Plus C18 analytical column (4.6 mm × 250 mm; particle size, 5 μm) was used for the separation. According to a method by Vranac [17], the binary mobile phase A was water, mobile phase B was methanol, and the gradient program was as follows: 20% B to 30% B in 0–5 min, 30% B to 70% B in 5–8 min, 70% B to 100% B in 8–18 min, and 100% B in 18–28 min. The flow rate was 1.0 mL/min for a total run time of 28 min. The injection volume was 10 μL for all samples. The detector was set at 280 nm for simultaneous monitoring of the different groups of phenolic compounds.

### 3.7. PPO Activity Determination

PPO activity was assayed according to the procedure described by Gu et al. [2]. One gram of pepper berries was homogenized with 10 mL of 0.1 M phosphate buffer for 15 min (pH 7) after frozen grinding. The supernatant was centrifuged (8000 r/min) at 4 °C for 20 min and then used to determine PPO activity.

### 3.8. Determination of Chlorophyll

Chlorophyll concentrations were determined according to Aronoff’s method, with modifications [18]. Samples of 0.5 g of pepper berries was extracted with 10 mL 80% acetone aqueous solution and ultrasonication (twice for 30 min each in the dark). The mixture was centrifuged for 20 min (8000 r/min). The solution was then immediately diluted to a volume of 25 mL with 80% acetone solution and mixed thoroughly. The content of chlorophyll a and chlorophyll b were measured by Arnon Formulas.

### 3.9. Determination of VC

The content of VC was analyzed by HPLC (Agilent 1260) according to the method previously described, with slight modification [19]. A sample of 2 g pepper berries was extracted by 2.5% metaphosphoric acid solution (30 mL) at 4 °C for 3 h and then centrifuged (12,000 rpm, 4 °C) for 10 min. The supernatant was filtered through a 0.45 μm filter. Separation was carried out on an Eclipse plus C18 (4.6 mm × 250 mm, particle size, 3.5 μm), at a temperature of 30 °C, a flow rate of 1 mL/min, and detection at 254 nm. An isocratic elution method with 95% KH_2_PO_4_ (25 mM) and 5% acetonitrile was used.

### 3.10. Statistical Analysis

Data were expressed as the mean ± standard deviation (SD). Analysis of variance (ANOVA) was performed, and comparisons of the means were carried out by least-significant difference (LSD) and Duncan. A value of *p* < 0.05 was considered statistically significant. Data were analyzed by using Origin 9 (OriginLab Corporation, Northampton, MA, USA) and SPSS Statistics 22 software packages (IBM Corporation, New York, NY, USA).

## 4. Conclusions

The activity of PPO and the contents of phenolic compounds, VC, and chlorophyll a and b in black pepper berries significantly decreased after the four different processing treatments. After the blanching treatment (80 °C for 120 s), PPO had poor heat resistance, and VC had poor stability. These substances also decreased during mild fermentation. In summary, the browning pathways in the preparation of black pepper mainly included enzymatic browning and non-enzymatic browning. PPO, phenolic substances, chlorophyll a and b, and VC were possibly involved in browning, and the complex reaction mechanism affected not only the formation of color but also the quality of pepper flavor. Therefore, the mechanism of browning was of great significance. Further separation of phenolic compounds and the study of chlorophyll derivatives should be conducted in the future. Pepper phenolic compounds were resolved by HPLC. The crude extracts of pepper phenolic compounds were obtained by the system solvent method. The extraction rates of phenolic compounds and the total phenolic content increased with increasing polarity of the extraction solvent. 

## Figures and Tables

**Table 1:** The four treatment methods of black pepper.

Methods	Processes
Direct sun drying	The berries were processed by sun-drying for five days. Sample S0 represents fresh pepper berries, Samples S1–S5 represent berries that underwent sun-drying for one to five days, respectively.
Blanching before sun drying	Fresh pepper berries were subjected to heat treatment with hot water for 2 min at 80 °C, and then the berries were processed by sun-drying for three days. Sample S0 represents fresh pepper berries. Sample C0 refers to berries subjected to heat treatment. Samples C1–C3 represent berries that underwent sun-drying for one to three days after heat treatment, respectively.
One-day sun drying before mild fermentation	Fresh pepper berries were subjected to heat treatment with hot water for 2 min at 80 °C. The berries were processed by sun-drying for one day. Then, they were fermented for one day before sun-drying. Samples F1-0 refer to berries that underwent sun-drying for one day after heat treatment and fermentation for one day. Samples F1-1 and F1-2 represent berries that underwent sun-drying for one or two days after fermentation, respectively.
Two-day sun drying before mild fermentation	Fresh pepper berries were subjected to heat treatment with hot water for 2 min at 80 °C, and then the berries were processed by sun-drying for two days. Then, they were fermented for one day before sun-drying. Samples F2-0 represent berries that underwent sun-drying for two days after heat treatment and fermentation for one day. Samples F2-1 and F2-2 refer to berries that underwent sun-drying for one or two days after fermentation, respectively.
